# Bariatric Surgery-Induced Telogen Effluvium (Bar SITE): Case Report and a Review of Hair Loss Following Weight Loss Surgery

**DOI:** 10.7759/cureus.14617

**Published:** 2021-04-21

**Authors:** Rena A Cohen-Kurzrock, Philip R Cohen

**Affiliations:** 1 Health Administration, Louisiana State University of Shreveport, Shreveport, USA; 2 Dermatology, San Diego Family Dermatology, National City, USA

**Keywords:** bariatric, effluvium, gastric, hair, loss, obesity, sleeve, surgery, telogen, weight

## Abstract

Bariatric surgery is a potential modality for the management of obesity. Bariatric patients may experience skin disorders and hair loss postoperatively. A 24-year-old woman with polycystic ovarian syndrome-associated obesity successfully underwent bariatric surgery. Within seven weeks after surgery, she developed diffuse and progressive hair loss, characteristic of telogen effluvium. Alopecia following bariatric surgery may be acute in onset, occurring within the first three months and often associated with telogen effluvium. In addition, bariatric surgery postoperative hair loss may be the result of nutritional deficiencies; in this setting, it is often chronic in onset, occurring six months after surgery. Also, hair loss in bariatric patients may be multifactorial in etiology. We introduced an acronym to facilitate the description of patients who experience bariatric surgery-induced telogen effluvium: Bar SITE.

## Introduction

Bariatric surgery is one of the treatment modalities available for the management of obese patients. The four common types of weight loss surgery include duodenal switch with biliopancreatic diversion, laparoscopic adjustable gastric banding, Roux-en-Y gastric bypass, and sleeve gastrectomy. Bariatric surgery not only can affect the skin, but also can cause or modify cutaneous diseases [1-15].

Telogen effluvium is a nonscarring type of alopecia that results in diffuse scalp hair loss approximately three months following a triggering cause. Surgery-induced telogen effluvium (SITE) results after various operations. Bariatric surgery-induced telogen effluvium (Bar SITE), with excessive hair loss resulting from the procedure-associated anagen follicles that prematurely progress to the telogen phase, has also occasionally been described [16-19].

A 24-year-old woman with polycystic ovary syndrome-associated obesity underwent bariatric surgery. Postoperatively, she developed telogen effluvium within three months. Hair loss in post-bariatric patients is reviewed.

## Case presentation

A 24-year-old woman had been diagnosed with polycystic ovary syndrome at age 12 years. Her medical management included metformin and an oral contraceptive (drospirenone/ethiny/estradiol/leromefolate calcium). However, she remained amenorrheic, continued to gain weight, and developed other syndrome-associated manifestations, such as acne and hirsutism.

Examination revealed an otherwise healthy woman whose weight was 208 pounds and whose height was 5 feet and 2 inches; her body mass index was 38 (normal, 18.5-24.9). Facial cutaneous examination was notable for her cheeks having inflammatory papules. Excessive hair was presented on her face, arms, legs, and chest.

Oral contraceptive therapy partially controlled her acne; laser hair removal had been performed on a monthly basis for her hirsutism. Despite oral medications, dietary restriction, and exercise, she was not able to maintain or lose weight. After consultation with a bariatric surgeon, she decided to undergo a gastric sleeve operation.

**Figure 1:**
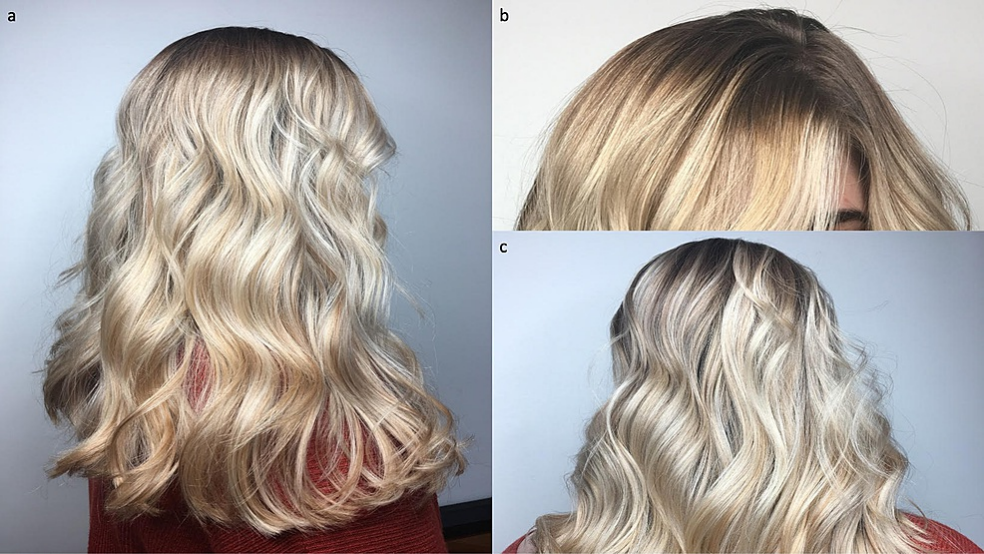
Scalp hair appearance prior to bariatric surgery Right side (a), frontal (b), and posterior (c) views of a 24-year-old woman show thick and dense scalp hair before undergoing a gastric sleeve operation for weight loss.

**Figure 2:**
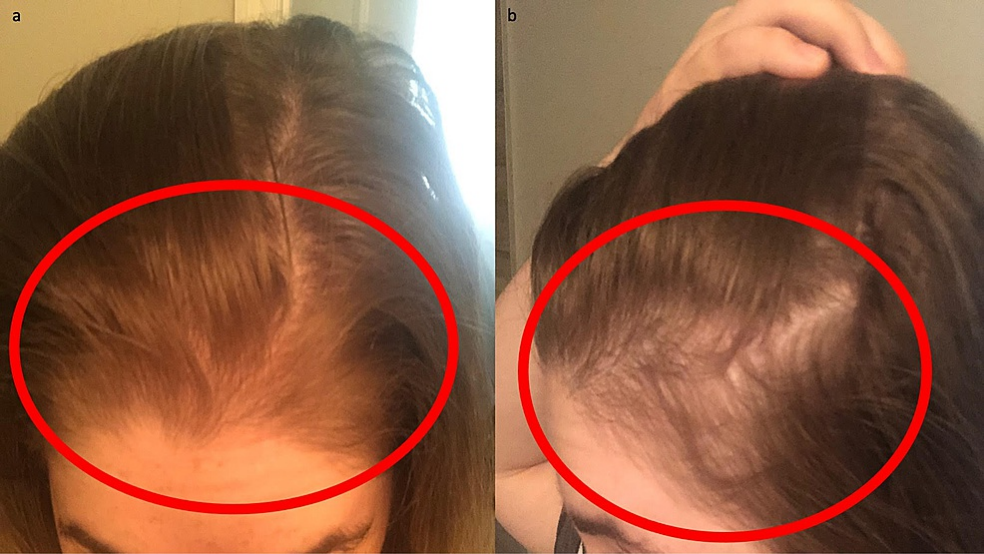
Bariatric surgery-induced telogen effluvium following gastric sleeve operation Frontal (a) and left side (b) views show extensive and diffuse hair loss (demonstrated within the red oval) secondary to telogen effluvium following bariatric surgery that was performed five months earlier.

**Figure 3:**
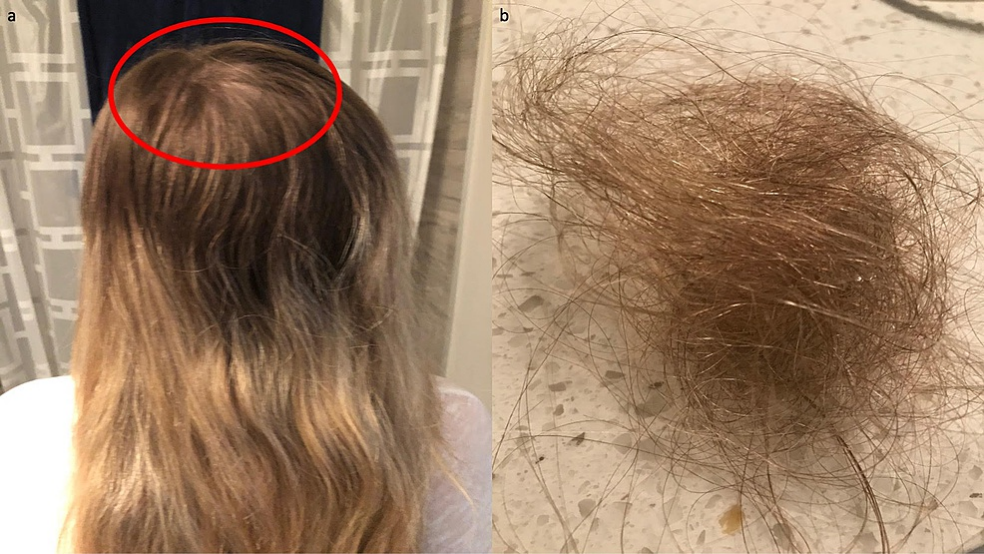
Bariatric surgery-induced telogen effluvium following gastric sleeve operation The posterior view (a), five months following bariatric surgery, shows pronounced hair loss on the crown of the scalp (demonstrated within the red oval). After a single brushing of the hair, significant shredding can readily be observed (b).

Prior to surgery, her scalp hair was thick and dense (Figure 1). However, seven weeks postoperatively, she began to notice diffuse hair loss. Her hair loss continued during the next nine months (Figure 2 and Figure 3).

Laboratory evaluation, including calcium, ferritin, folate, iron, and vitamins (A, B1, B6, B12, and D 25-hydroxy), was performed. The studies did not reveal any abnormalities. Follow-up clinical examination, 11 months postoperatively, demonstrated that both the rate and the extent of her hair loss had decreased.

**Figure 4:**
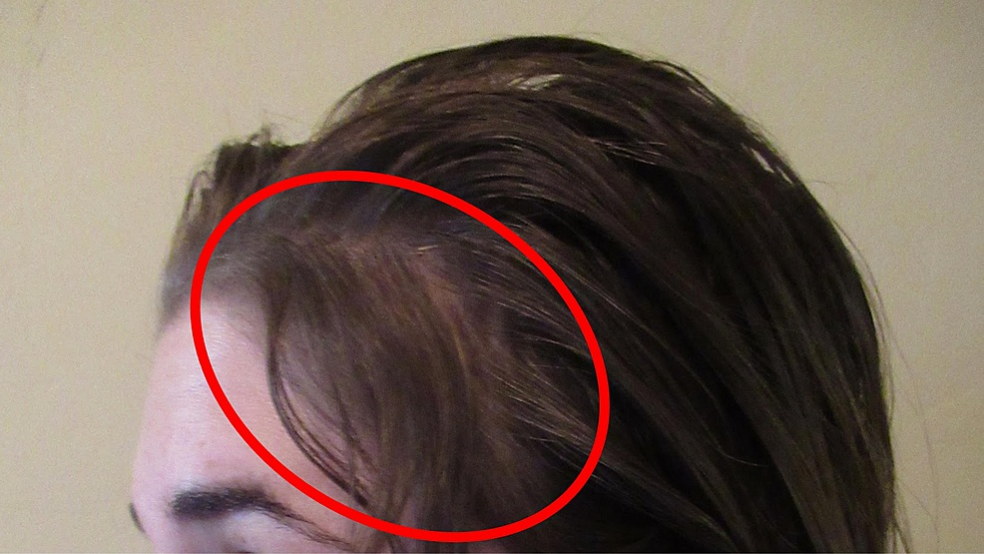
Resolution of bariatric surgery-induced telogen effluvium The left-side view of the patient’s scalp shows resolution of telogen effluvium with near-complete hair growth (demonstrated within the red oval) 14 months following gastric sleeve operation for weight loss.

Correlation of her history and clinical presentation established the diagnosis of telogen effluvium. The bariatric surgery was the associated etiologic event that triggered her hair loss. Nutritional deficiency-associated hair loss was excluded by her normal laboratory studies. Her alopecia resolved and there was significant new hair growth within 14 months after surgery (Figure 4).

## Discussion

Bariatric surgery can affect preexisting skin conditions or result in new cutaneous disorders or both. Hidradenitis suppurativa and psoriasis can remain the same, worsen, or improve after bariatric surgery. Diabetic skin changes, such as acanthosis nigricans and necrobiosis lipoidica, can improve [1,3,7].

Bariatric surgery can also be associated with the development of new skin conditions. Bowel-associated dermatitis-arthritis syndrome (a neutrophilic dermatosis with features of arthralgias and arthritis, fever, myalgias, tenosynovitis, and skin lesions, such as erythema nodosum, macular ecchymoses, nodular erythematous plaques, papules, and/or pustular vasculitis) can occur three months to five years following bariatric surgery. It can present following either intestinal bypass surgery or sleeve gastrectomy. Less common disorders include angiosarcoma, dermatitis herpetiformis, and vasculitis (such as Henoch-Schonlein purpura) [4-6,8].

Nutritional deficiencies, some of which are associated with dermatoses, occur in about half of the bariatric surgery patients. They can result from decreased levels of copper, essential fatty acid, iron, protein, selenium, vitamins (A, B1, B12, C, E, folate, and K), and zinc (acquired acrodermatitis enteropathica). They occur more frequently after malabsorptive surgical procedures (biliopancreatic diversion with duodenal switch, malabsorptive surgery, and Roux-en-Y gastric bypass) than restrictive operations (adjustable gastric band and laparoscopic sleeve gastrectomy). Life-long vitamin supplementation after surgery should include calcium, multivitamin with minerals, vitamin B12, and vitamin D. Additionally, other vitamins and trace elements for supplementation may include elemental iron, folic acid, selenium, vitamin A, and zinc [2,4].

There are several etiologies for hair loss in women; they can be classified as being associated with either the presence or absence of scarring. Common causes of nonscarring alopecia in women include androgenetic alopecia, alopecia areota, telogen effluvium, and nutritional deficiency. Telogen effluvium has several potential precipitating factors; stress associated with a major surgery is one of its causes [16-19].

Alopecia is not uncommon after bariatric surgery. It has been observed after the following procedures: laparoscopic adjustable gastric banding, laparoscopic sleeve gastrectomy, Roux-en-Y gastric bypass, and vertical banded gastroplasty; to the best of our knowledge, a study evaluating the incidence of alopecia associated with each of these procedures has not been performed. The hair loss can be either acute onset within the first three or four postoperative months or chronic onset, beginning after six months [9-15].

Acute-onset alopecia following bariatric surgery is usually caused by telogen effluvium; similar to telogen effluvium caused by other etiologies, the management of bariatric surgery-induced telogen effluvium includes clinical monitoring of subsequent hair growth. In contrast, hair loss that is chronic in onset following weight loss surgery is often associated with a nutritional deficiency; preliminary laboratory studies to evaluate for nutrition deficiency includes calcium, ferritin, folate, iron, and vitamins (A, B1, B6, B12, and D 25-hydroxy). However, hair loss in bariatric patients can be multifactorial and caused by more than one etiology [1-4,9-15].

In addition to telogen effluvium and nutritional deficiencies, laboratory evaluation may be helpful to exclude other causes of hair loss in women following bariatric surgery. Preliminary studies may include complete blood count, comprehensive serum chemistries, dehydroepiandrosterone sulfate, ferritin, follicle-stimulating hormone, iron, luteinizing hormone, testosterone (free and total), thyroid panel (free triiodothyronine, free thyroxine, and thyroid-stimulating hormone), total iron binding capacity, and prolactin. Additional studies may include 17-hydroxyprogesterone, antinuclear antibody, rapid plasma reagin, and vitamin D [16,17].

We introduce the acronym Bar SITE (bariatric surgery-induced telogen effluvium). The word “Bar” is the first three letters of “Bariatric” and the word “SITE” originates from the first letters of each of the following words, “Surgery-Induced Telogen Effluvium.” We anticipate that the use of this acronym may aid clinicians when describing patients with hair loss attributed to this etiology following obesity-associated weight loss surgery.

## Conclusions

Bariatric surgery is associated with dermatologic disorders, including hair loss. Telogen effluvium is a nonscarring alopecia, which may occur following bariatric surgery and usually presents within three months after surgery. However, alopecia after bariatric surgery can also result from nutritional deficiency, which usually presents several months following surgery. Bar SITE is a useful acronym to describe bariatric surgery-induced telogen effluvium.
